# Repair of Oxidative DNA Base Damage in the Host Genome Influences the HIV Integration Site Sequence Preference

**DOI:** 10.1371/journal.pone.0103164

**Published:** 2014-07-22

**Authors:** Geoffrey R. Bennett, Ryan Peters, Xiao-hong Wang, Jeungphill Hanne, Robert W. Sobol, Ralf Bundschuh, Richard Fishel, Kristine E. Yoder

**Affiliations:** 1 Molecular Virology, Immunology, and Medical Genetics, The Ohio State University Wexner Medical Center and Comprehensive Cancer Center, The Ohio State University, Columbus, Ohio, United States of America; 2 Department of Pharmacology & Chemical Biology, University of Pittsburgh School of Medicine, Pittsburgh, Pennsylvania, United States of America; 3 University of Pittsburgh Cancer Institute, Hillman Cancer Center, Pittsburgh, Pennsylvania, United States of America; 4 Department of Human Genetics, University of Pittsburgh Graduate School of Public Health, Pittsburgh, Pennsylvania, United States of America; 5 Department of Physics, The Ohio State University, Columbus, Ohio, United States of America; 6 Department of Chemistry and Biochemistry, Division of Hematology, Department of Internal Medicine, Center for RNA Biology, The Ohio State University, Columbus, Ohio, United States of America; University of Iowa, United States of America

## Abstract

Host base excision repair (BER) proteins that repair oxidative damage enhance HIV infection. These proteins include the oxidative DNA damage glycosylases 8-oxo-guanine DNA glycosylase (OGG1) and mutY homolog (MYH) as well as DNA polymerase beta (Polβ). While deletion of oxidative BER genes leads to decreased HIV infection and integration efficiency, the mechanism remains unknown. One hypothesis is that BER proteins repair the DNA gapped integration intermediate. An alternative hypothesis considers that the most common oxidative DNA base damages occur on guanines. The subtle consensus sequence preference at HIV integration sites includes multiple G:C base pairs surrounding the points of joining. These observations suggest a role for oxidative BER during integration targeting at the nucleotide level. We examined the hypothesis that BER repairs a gapped integration intermediate by measuring HIV infection efficiency in *Polβ* null cell lines complemented with active site point mutants of *Polβ*. A DNA synthesis defective mutant, but not a 5′dRP lyase mutant, rescued HIV infection efficiency to wild type levels; this suggeted Polβ DNA synthesis activity is not necessary while 5′dRP lyase activity is required for efficient HIV infection. An alternate hypothesis that BER events in the host genome influence HIV integration site selection was examined by sequencing integration sites in *OGG1* and *MYH* null cells. In the absence of these 8-oxo-guanine specific glycosylases the chromatin elements of HIV integration site selection remain the same as in wild type cells. However, the HIV integration site sequence preference at G:C base pairs is altered at several positions in *OGG1* and *MYH* null cells. Inefficient HIV infection in the absence of oxidative BER proteins does not appear related to repair of the gapped integration intermediate; instead oxidative damage repair may participate in HIV integration site preference at the sequence level.

## Introduction

**Figure 1:**
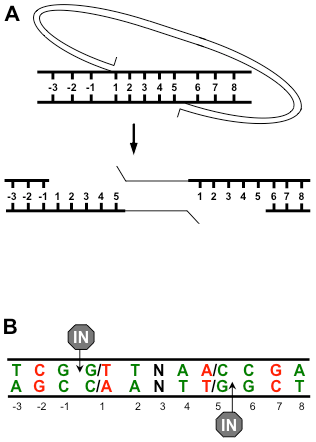
Retroviral integration. *(A)* Viral cDNA is depicted by a thin line and host target DNA is indicated by a thick line. Base pairs in the host target DNA are numbered. The HIV LTR ends are covalently joined to the target DNA 5 base pairs apart. The intervening host DNA denatures yielding an integration intermediate with two 5 base pair gaps. *(B)* The sequence preference observed at HIV integration sites. The numbering is identical to (A) and the points of joining are indicated by “IN”. Base pairs in green are favored and base pairs in red are disfavored at HIV integration sites.

Retroviruses are defined by two enzymatic activities, reverse transcription and integration [1]. The viral enzyme reverse transcriptase copies the viral genomic RNA to a linear double stranded cDNA. The cDNA is part of a pre-integration complex (PIC) that is poorly understood. The PIC travels to the nucleus where the viral protein integrase mediates the covalent joining of the viral cDNA to the host chromosome. The two ends of the viral cDNA are joined to the host DNA 4–6 base pairs apart, depending on the retrovirus. In the case of HIV, 5 base pairs separate the points of joining, the distance across one major groove (Figure 1A). The process of integration yields an integration intermediate comprised of the viral cDNA flanked by 5 base gaps of host sequence and 5′ dinucleotide flaps of viral sequence. Host enzymes are assumed to repair this integration intermediate, but identities of the specific repair proteins remain unknown [2].

The host protein PSIP1/LEDGF p75 is the major host co-factor for HIV integration [3]–[7]. This protein specifically binds HIV integrase with an integrase-binding domain (IBD) and binds to chromatin via a PWWP domain and two AT hook motifs [8]–[16]. Studies of *LEDGF* depletion and deletion cells have shown that LEDGF is required for efficient integration to chromatin *in vivo* [4], [5]. LEDGF also enhances integration by recombinant integrase *in vitro* [17]–[19].

Integration sites from multiple retroviruses have been sequenced and mapped in relation to chromatin features such as transcription units, promoters, CpG islands, and local G:C content [5], [20]–[23]. Retroviruses have distinct integration site preferences. For example, HIV preferentially integrates to transcription units but does not favor promoters while MLV favors integration near promoter sequences. The sequence at the site of integration, including the 4–6 base pairs between and up to three base pairs flanking the points of joining, show a palindrome of preferred and disfavored nucleotides (Figure 1B, [24], [25]). Sequences from cytoplasmic HIV PICs integrated to a naked DNA target showed that the central 5 base pairs retained the sequence preference as well as positions −3 and 6 [25]. This comparison suggests that many base pairs at the integration site are recognized by integrase, but additional host factors may also influence integration at some flanking nucleotides. While deletion of *LEDGF* leads to significant differences of integration targeting to genomic features *in vivo*, it had no effect on the palindromic sequence preference [5], [26].

The short patch oxidative base excision repair (BER) pathway has been implicated in the integration of HIV [27], [28]. The BER pathway recognizes damage on individual bases, including deamination, alkylation, and oxidation [29]–[31]. Glycosylases recognize specific damages of DNA bases. The damaged base is removed by a glycosylase leaving an abasic site. The essential enzyme apurinic/apyridimic endonuclease (APEX1) recognizes the abasic site and cleaves the DNA backbone at the 5′ side of the lesion to generate a 3′ hydroxyl and a 5′ deoxyribose phosphate (5′dRP) flap. In the short patch BER pathway, DNA polymerase β (Polβ) synthesizes one base and also removes the 5′dRP lesion. Repair is completed when the remaining nick is ligated by a heterodimer of Lig3 and XRCC1 or via LigI [32]. Proteins throughout the pathway from oxidative base damage recognition through the final ligation step were identified in an siRNA screen for DNA repair factors affecting HIV infection [27]. Reduced expression of oxidative BER genes led to a decrease of HIV infection efficiency. The absence of BER proteins was characterized to reduce HIV integration [27], [28].

The mechanism of BER mediated effects on HIV integration has not been shown. One formal possibility is repair of the gapped integration intermediate by BER. We have further explored the effects of BER proteins on HIV integration with well-defined BER mutant cell lines. The previously observed effects of the BER pathway on HIV integration do not appear related to gap repair of an integration intermediate. Instead, oxidative glycosylases with specific base recognition may affect the sequence preference of HIV integration. This suggests that integration may be favored at sites of oxidative DNA repair intermediates.

## Materials and Methods

### Cell lines

Matched wild type and *Polβ* null murine embryonic fibroblasts were derived from littermates and have been previously described [33]. The *Polβ* null cell line was stably transfected with an empty vector (Polβ−/− Neo) or the same vector expressing the wild type *Polβ* cDNA (PolB−/− +PolB), a polymerase defective *Polβ(D256A)* mutant cDNA (PolB−/− +PolB (*pol*-)), a lyase defective *Polβ(K35,68,72A)* mutant cDNA (PolB−/− +PolB (*lyase*-)), or a completely inactive *Polβ(K35,68,72A, D256A)* mutant cDNA (PolB−/− +PolB (*pol*-*lyase*-)) [33]. Nuclear extracts were used to determine the protein expression of the endogenous and transgenic Polβ proteins in established stable cell lines. Nuclear extracts were prepared with the NucBuster protein extraction kit and protein concentration was determined as described previously. 20–30 µg nuclear extract proteins were loaded on a precast 4–12% NuPAGE Tris-glycine gel, run 2–3 h at 100 volts. The gel was transferred to a 0.45 µm nitrocellulose membrane (Bio-Rad) at 0.2 mA for 2–3 hours. The membrane was blotted with anti-Polβ (1∶5000, Clone 18S) or anti-PCNA (1∶2000, SC-56, Santa Cruz Biotechnology), followed by the secondary antibody Immun-Star goat anti-mouse HRP conjugate (Bio-Rad). After washing, the membrane was illuminated with Immun-Star HRP peroxide buffer with luminol/enhancer (Bio-Rad).

Matched wild type, *OGG1* null, and *MYH* null murine embryonic fibroblasts were also derived from littermates and have been previously described [28], [34]. All cells were grown in DMEM supplemented with 10% fetal bovine serum, GlutaMAX glutamine, and Penicillin-Streptomycin (Gibco, Life Technologies). The media of transfected *Polβ* null cell lines was supplemented with 600 µg/ml geneticin. Cells were grown at 37°C with 10% CO_2_.

### HIV vector particles

**Figure 2:**
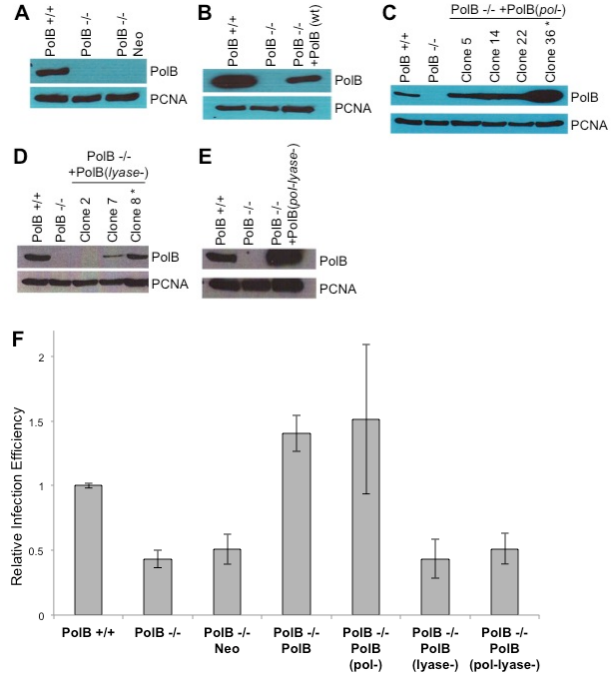
Polβ polymerase activity is not required for efficient HIV infection. Murine embryonic fibroblasts with a deletion of the Polβ cDNA (PolB−/−) were complemented with *(A)* an empty vector (Neo), *(B)* the wild type cDNA (PolB), *(C)* a polymerase defective point mutant gene (PolB (pol-)), *(D)* a lyase deficient mutant cDNA (PolB (lyase-)), or *(E)* an enzymatically dead mutant (PolB (pol-lyase-)). *(F)* These cell lines and a matched wild type cell line were infected with an HIV based vector expressing GFP following integration. Cells were analyzed by flow cytometry for GFP expression, an indicator of successful infection efficiency. Results are from three independent experiments of duplicates and are expressed relative to wild type cells. Error bars indicate the standard deviations.

**Figure 3:**
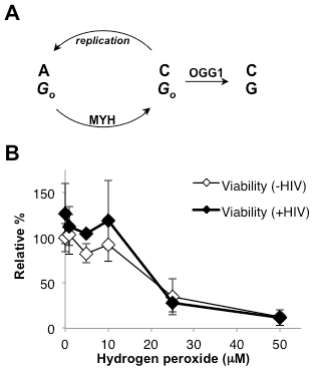
Oxidative damage during HIV infection. *(A)* OGG1 and MYH both recognize 8-oxo-dG damage (shown as *G_o_*). OGG1 repairs 8-oxo-dG to G. Replication of 8-oxo-dG results in an 8-oxo-dG:A mispair, which is recognized by MYH. The MYH glycosylase initiates repair of the mispaired A to C yielding an 8-oxo-dG:C base pair. The product of the MYH repair reaction must still be repaired by OGG1. *(B)* Wild type cells were infected with an HIV based vector expressing GFP. Cells were treated with increasing concentrations of H_2_O_2_ immediately prior to infection. Viability in the absence (open diamonds) or presence of HIV (closed diamonds) was measured by trypan blue exclusion. Error bars indicate the standard deviations from three independent experiments.

HIV lentiviral vectors expressing GFP were generated by triple transfection of human fibroblast 293T cells with a packaging construct ΔR9, a viral genomic RNA plasmid p156RRLsinPPTCMVGFPPRE, and a plasmid expressing the envelope gene *VSV-G* [35], [36]. Supernatants containing vector particles were sterile filtered to remove producer cells and treated with DNaseI to digest producer plasmids. Target cells were plated in 6 well dishes at a density to achieve 2×10^5^ cells 24 hours after plating. Cell density was verified by counting with trypan blue (Gibco, Life Technologies). Vector particles were added to target cell media in the presence of 10 µg/ml DEAE-Dextran (Sigma Aldrich). The MOI of wild type Polβ cells in Figure 2 was 0.4 and in Figure 3 was 0.3. Cells were analyzed for infection efficiency 72 hours post infection by fixing with 4% paraformaldehyde (Sigma Aldrich) and scanning for GFP expression with a FACScalibur (BD Biosciences). Flow cytometry data was analyzed with FlowJo software. Cells were exposed to varying concentrations of H_2_O_2_ (Sigma Aldrich) in PBS for one hour at 37°C prior to infection. Viability was measured by trypan blue exclusion at 72 hours post infection.

### Integration site sequencing

Cells were infected at a 0.8 multiplicity of infection and grown for 7 days. Genomic DNA was harvested by DNeasy (Qiagen) and prepared for sequencing as described [37], [38]. All sequences had perfect homology to the terminal 18 bp of HIV U3 and mouse sequences were at least 20 bp with >98% identity. Sequences were mapped to the mouse genome using BLAT [39]. The number of individual integration sites analyzed included 240 from untreated wild type cells (GenBank Accession numbers KG960523–KG960762), 105 from wild type cells treated with 10 µM H_2_O_2_ (GenBank Accession numbers KG960306–KG960410), 112 from wild type cells treated with 30 µM H_2_O_2_ (GenBank Accession numbers KG960411–KG960522), 245 from *OGG1* null cells (GenBank Accession numbers KG960986–KG961230), and 223 from *MYH* null cells (GenBank Accession numbers KG960763–KG960985). The frequency of G or C in these murine cells is 0.205 and the frequency of A or T is 0.295. The observed frequencies in integration sites are expressed as the difference from random frequencies 0.205 or 0.295. Statistical significance of deviations of base frequency was determined by a two-tailed binomial test in Microsoft Excel. Statistical analysis of integration sites compared to genomic elements was by Fisher’s exact test using GraphPad Prism version 4.0 for Macintosh (GraphPad Software).

## Results

### Polβ 5′dRP lyase activity, but not polymerase activity, is necessary for efficient HIV infection

An HIV integration intermediate may be repaired *in vitro* by any combination of a polymerase, flap endonuclease, and ligase [2]. The host DNA repair polymerase Polβ could repair a model HIV integration intermediate *in vitro* by synthesizing nascent DNA at the gap. Single deletion of oxidative BER genes *OGG1*, *MYH*, or *Polβ* reduces HIV infection to approximately 40% of wild type cells [28]. *Polβ* null murine embryonic fibroblasts were complemented with an empty vector or the wild type *Polβ* cDNA (Figure 2A, B) [33]. The cells were infected with an HIV based retroviral vector expressing GFP following successful integration [36]. Infection efficiency was measured by flow cytometry of GFP positive cells. Complementation with the wild type *Polβ* cDNA, but not the empty vector, completely rescued infection efficiency (Figure 2F, *Polβ−/−* compared to *Polβ−/−* complemented with an empty expression vector p = 0.91, *Polβ−/−* compared to *Polβ−/−* complemented with a wild type *Polβ* transgene p<0.0001).

Polβ has two distinct enzymatic activities which may be distinguished by mutations at two separate active sites [33]. In addition to polymerase activity, the lyase activity of Polβ cleaves a 5′dRP flap that occurs during BER. DNA synthesis activity is abolished by the mutation Polβ(D256A); a triple mutation Polβ(K35A/K68A/K72A) abrogates the 5′dRP lyase activity [33]. *Polβ* null cells were complemented with mutant transgenes affecting only the polymerase or the 5′dRP lyase active site (Figure 2C, D). These cells were infected with an HIV based retroviral vector expressing GFP and assayed by flow cytometry. Complementation with a polymerase defective, 5′dRP lyase active *Polβ* transgene was able to rescue HIV infection efficiency to wild type levels (Figure 2F, *Polβ−/−* compared to *Polβ−/−* complemented with a *Polβ(D256A)* transgene p = 0.01). However, a polymerase active, 5′dRP lyase defective transgene was unable to rescue HIV infection efficiency (Figure 2F, *Polβ−/−* compared to *Polβ−/−* complemented with a *Polβ(K35A/K68A/K72A)* transgene p = 0.21). This data suggests that the polymerase activity of Polβ is not necessary for HIV integration and does not support a model for BER repair of a gapped integration intermediate. The data does indicate that the 5′dRP lyase activity of Polβ is necessary for efficient integration. A combination mutant protein defective for both 5′dRP lyase and polymerase activities was also unable to rescue the HIV infection phenotype (Figure 2E, F, *Polβ−/−* compared to *Polβ−/−* complemented with a *Polβ(D256A, K35A/K68A/K72A)* transgene p = 0.14). The presence of enzymatically inactive Polβ protein is not sufficient to support HIV integration.

### BER proteins and oxidative damage influence HIV integration site sequence preference

If the oxidative BER pathway is not repairing the integration intermediate, it must enhance integration by an alternative mechanism. The most common form of oxidative DNA base damage is 8-oxo-guanine (8-oxo-dG) [29]. The sequence preference at HIV integration sites includes multiple G:C base pairs (Figure 1B). This observation suggested that sites of oxidative DNA base repair might influence HIV integration; the sequence preference at integration sites could be 8-oxo-dG:C base pairs, which are not detectable by sequencing integration sites. Of the oxidative BER proteins, OGG1 and MYH have specificity for only 8-oxo-dG [30]. OGG1 directly recognizes 8-oxo-dG and initiates repair by removing the damaged base (Figure 3A). During replication 8-oxo-dG mispairs with A, resulting in an 8-oxo-dG:A mismatch. MYH specifically recognizes and binds both bases in this mismatch, but does not remove the damaged base. Instead MYH excises the mispaired A and leaves the damaged 8-oxo-dG, allowing a subsequent round of repair by OGG1 that will remove the 8-oxo-dG (Figure 3A) [40]. The remaining BER proteins, including Polβ, participate in repair of multiple oxidatively damaged bases.

**Figure 4:**
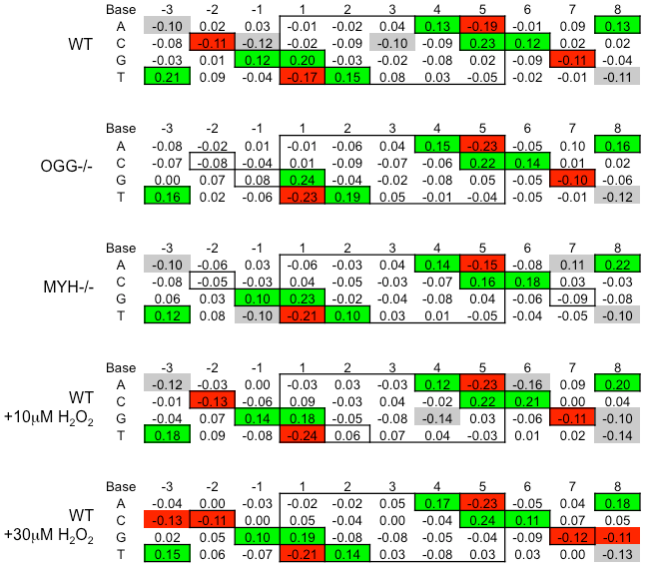
Effects of 8-oxo-dG specific glycosylases or hydrogen peroxide on HIV integration site sequence preference. Wild type, *OGG1* null, *MYH* null, and wild type cells treated with 10 µM or 30 µM H_2_O_2_, were infected with an HIV based retroviral vector at 0.8 MOI. 10 µM H_2_O_2_ is less than the 50% lethal dose for the wild type cells and 30 µM H_2_O_2_ is greater. After 7 days, genomic DNA was purified. HIV integration sites were subcloned, sequenced, and mapped to the mouse genome. The random frequency of G or C in the mouse genome is 0.205 and A or T is 0.295. The differences in observed base frequencies relative to the random frequencies are shown. Base numbering relative to the HIV points of joining is as in Figure 1. Boxes indicate the known HIV integration base preferences for wild type cells. Green, red, and gray highlights indicate a statistically significant difference of >0.10 from random frequency with a p value of <0.005. Green highlights are positive differences and red highlights are negative previously published palindromic prefered bases. Deletion of the *OGG1* gene leads to a loss of HIV sequence preference at positions −2 and −1. Deletion of the *MYH* gene also leads to loss of the HIV sequence preference at positions −2 and 7. While treatment of wild type cells with 10 µM H_2_O_2_ did not dramatically alter the sequence preference at integration sites, treatment with 30 µM H_2_O_2_ led to the disfavor of C at position −3 and disfavor of G at position 8, highlighted in red.

Murine embryonic fibroblasts with deletions of *OGG1* or *MYH* and matched wild type cells were infected with an HIV based retroviral vector. The genomic DNA of infected cells was purified, integration sites were subcloned, sequenced, and mapped to the mouse genome [37]. The sequence preference of integration sites from wild type cells was similar to that previously observed during HIV infection (Figure 4, [24], [25]). In the absence of OGG1 or MYH there was no change of the integration site base preference within the central 5 bp duplication.

In *OGG1* null cells integration sites showed a loss of any statistically significant preference for G at position −1 or C at position −2 (Figure 4). In *MYH* null cell lines there is a loss of base preference for C at positions −2 and 7. Although C is not recognized by either repair protein, MYH or OGG1 would recognize 8-oxo-dG on the opposite strand (Figure 3A). Thus the deletion of 8-oxo-dG specific glycosylase genes *MYH* and *OGG1* specifically alters the HIV integration site preference at G:C base pairs near the points of joining. These changes are reminiscent of the HIV PIC integration sequence preference *in vitro* which also loses preference for G:C base pairs at positions −2, −1, and 7 [25].

Wild type cells were also treated with the oxidative damage-inducing agent H_2_O_2_ immediately before HIV infection. Treatment of wild type cells with 10 µM H_2_O_2_ has no effect on viability, but treatment with 25 µM H_2_O_2_ reduces viability to less than 40% (Figure 3B). Thus 10 µM H_2_O_2_ is less than and 25 µM H_2_O_2_ is greater than the 50% lethal dose in this cell line. The sensitivity of these wild type cells to H_2_O_2_ is not affected by HIV infection (Figure 3B). The major base damage induced by H_2_O_2_ is on guanines [41]. The integration site sequence preference for 10 µM or 30 µM H_2_O_2_ was similar to untreated wild type cells (Figure 4). However, the sequence preference observed in 30 µM H_2_O_2_ treated cells was expanded to the −3 and 8 positions. While T and A continue to be favored at positions −3 and 8, respectively, H_2_O_2_ treatment led to a statistically significant disfavor of C at position −3 and G at position 8 (Figure 4). Increased oxidative damage at the time of infection appears to augment the HIV integration site sequence preference.

**Figure 5:**
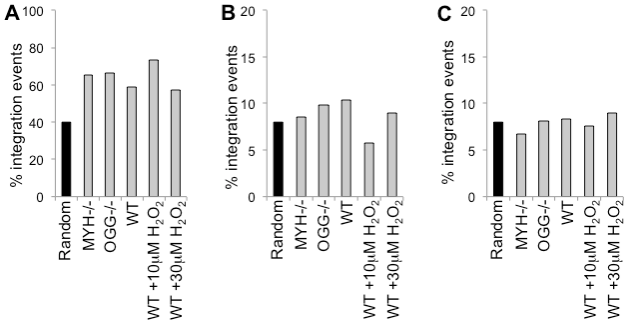
HIV integration near genomic elements. HIV integration sites were mapped to genomic elements in *OGG1* null cells, *MYH* null cells, untreated wild type cells, and wild type cells treated with 10 µM or 30 µM H_2_O_2_. (**A**) HIV has a preference for integration to transcription units. (**B**) HIV shows no preference for integration to promoters. (**C**) There is no preference for HIV integration within 5 kb of CpG islands.

**Figure 6:**
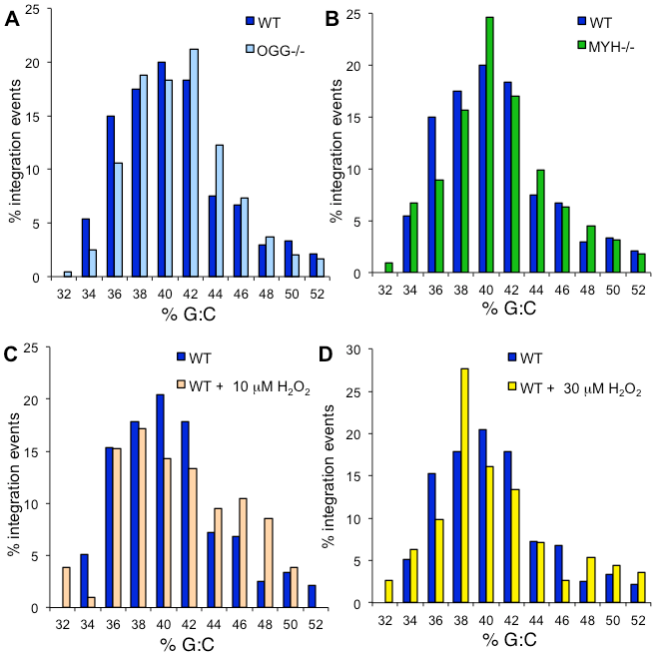
G:C content surrounding HIV integration sites. The percentage of HIV integration sites compared to the percentage of G:C base pairs within a 5 kb window is shown for wild type cells compared to (**A**) *OGG1* null cells, (**B**) *MYH* null cells, (**C**) wild type cells treated with 10 µM H_2_O_2_, and (**D**) wild type cells treated with 30 µM H_2_O_2_. The frequency of G:C base pairs in the mouse genome is 0.41. There is no significant difference between HIV integration sites in untreated wild type cells and *OGG1* null (p = 0.96), *MYH* null (p = 0.99), 10 µM H_2_O_2_ treated (p = 0.67), or 30 µM H_2_O_2_ treated cells (p = 0.88).

Integration sites were also analyzed for their proximity to genomic level elements. HIV shows a preference for integration in transcription units, but not near promoters. Deletion of the known HIV integrase co-factor LEDGF dramatically changes the integration preference for these elements [5]. The integration sites from all of these cell lines or wild type cells treated with H_2_O_2_ showed a preference for integration to transcription units (Figure 5A, p<0.05 for all cell lines), but no preference for integration near promoters or CpG islands (Figure 5B and C, p>0.2 for all cell lines at both promoters and CpG islands). Deletion of LEDGF significantly alters the integration profile of G:C content surrounding the integration sites [5]. The profiles of G:C content within 5 kb around the integration sites are similar for all the BER cell lines (Figure 6). Thus the chromatin markers of integration sites are not affected by deletion of *OGG1* or *MYH* or treatment with H_2_O_2_.

## Discussion

DNA repair proteins throughout the oxidative BER pathway were identified in an siRNA screen for factors affecting HIV infection [27]. Other members of the BER pathway that mediate repair of methylation, alkylation, or deamination base damage had no effect on HIV infection efficiency. Oxidative damage of DNA is always present in cells and BER proteins are expressed throughout the cell cycle [42], [43]. Presence of oxidative damage and oxidative DNA damage repair proteins appear to correlate with successful HIV infection.

One obvious mechanism for the role of a DNA repair pathway during HIV infection could be the repair of the gapped integration intermediate. However, several observations argue against this hypothesis. First, deletion of BER proteins does not affect infection efficiency of a gamma retrovirus [28]. It seems probable that the DNA repair pathway responsible for repair of integration intermediates would broadly repair this structure for all retroviruses. Second, there is no obvious role for glycosylases during gap repair. Finally, the polymerase activity of Polβ has no effect on HIV infection efficiency (Figure 2). Instead the 5′dRP lyase activity of Polβ appears to be important. The Polβ 5′dRP lyase active site is not likely able to accommodate the 5′ dinucleotide flap present in the HIV integration intermediate [44], [45]. A previous study of this repair event shows that wild type recombinant Polβ was not able to digest the 5′ dinucleotide flap of a model gapped integration intermediate *in vitro* [2]. Thus the role of the Polβ 5′dRP lyase activity is also not likely part of the integration gap repair event. While oxidative BER proteins may participate in repair of retroviral gapped integration intermediates, they are not absolutely required; there are likely multiple redundant proteins that participate in this repair [2]. Instead it appears that oxidative BER proteins identified in an siRNA screen may directly affect integration targeting at the sequence level.

The host protein LEDGF has been shown to directly bind the HIV integration complex and direct integration to sites within chromatin [46]–[48]. However, LEDGF has no effect on the sequence preference at the site of integration [5], [49], [50]. Deletion or reduced expression of the *LEDGF* gene leads to significantly decreased HIV infection [4], [5]. However, cytoplasmic or nuclear pre-integration complexes from *LEDGF* null cells show integration efficiency to a naked DNA target equal to PICs from wild type cells [5]. PICs from BER null cells show reduced integration efficiency to naked DNA targets compared to PICs from wild type cells, which can be rescued by addition of recombinant Polβ protein [28]. LEDGF appears to mediate HIV integration by tethering the PIC to chromatin, but not naked DNA [18]; BER proteins appear able to affect HIV integration targeting in naked DNA. Thus LEDGF affects HIV integration targeting on a chromatin scale, while BER proteins may affect integration targeting on a nucleotide scale.

One model for BER protein effects on targeting HIV integration is for direct protein binding with the integration complex. This model seems unlikely due to known binding of LEDGF to integrase and steric interference [46]–[48], [51]–[54]. Alternatively, the oxidative BER proteins may affect the DNA or chromatin in a way that enhances HIV integration efficiency. Oxidative damage of genomic DNA is constant and BER proteins are constitutively expressed [42], [43]. Glycosylases that do not recognize oxidative damage were shown to have no effect on HIV infection efficiency [27]. A major difference between oxidative glycosylases and all other glycosylases is their DNA product. All glycosylases remove a base leaving an abasic site. Oxidative glycosylases have AP (apurinic or apyridimic) lyase activity which nicks the sugar phosphate backbone at the abasic site. MYH is the only oxidative damage glycosylase that does not have AP lyase activity; however, MYH must always act in concert with OGG1 which does have AP lyase activity (Figure 3A). Hence, all oxidative base excision repair will result in an abasic site with a nick. HIV infection efficiency is reduced in the absence of glycosylases that have AP lyase activity or in the absence of Polβ 5′dRP lyase activity ([27] and this work).

An alternative model for the role of oxidative BER proteins during HIV integration is based on the nature of the oxidative BER DNA intermediates. In this model BER single base gap DNA intermediates, rather than the BER proteins, are the true mediators of enhanced HIV integration. The single base gap of short patch BER increases the local flexibility of DNA more than a nick or an abasic site [55]–[57]. HIV integration is enhanced by an obtuse angle of bent DNA and inhibited in an acute angle [58]. Thus HIV strand joining may be favored on a helical strand with local oxidative base repair (such as positions −1 and 6) and disfavored on the opposite strand (such as positions −3, −2, 7 and 8). The base preference of HIV integrase is subtle, requiring analysis of many integration sites. The observed differences in sequence preference are also subtle due in part to the apparent inherent promiscuity of HIV integrase as well as the redundancy of BER pathway proteins. For example, OGG1 only recognizes oxidatively damaged guanine, but other glycosylases also recognizes damaged guanine.

Additional studies are required to elucidate the roles of DNA lyase activity during HIV integration. Only glycosylases with AP lyase activity enhance HIV infection efficiency. While it is impossible to isolate the lyase activity of bifunctional glycosylases, it is possible to differentiate the DNA synthesis and 5′dRP lyase activities of Polβ. Whether the AP lyase activity of glycosylases and the 5′dRP lyase activity of Polβ play the same function during integration also requires further investigation. However, this data suggest the intriguing possibility that DNA lyase activity may be a novel enzymatic target for anti-retroviral therapies.

## Conclusions

HIV integration has a preference for both nucleotide sequence and chromatin features. The host protein LEDGF targets HIV to integrate near genomic elements but has no effect on the consensus sequence preference of integration sites. The oxidative BER pathway may influence the sequence preference at HIV integration sites. Additionally, host DNA lyase activity is a potential target for novel HIV inhibitors.
